# Condensed Tannins, a Viable Solution To Meet the Need for Sustainable and Effective Multifunctionality in Food Packaging: Structure, Sources, and Properties

**DOI:** 10.1021/acs.jafc.1c07229

**Published:** 2022-01-14

**Authors:** Lucia Panzella, Alessandra Napolitano

**Affiliations:** Department of Chemical Sciences, University of Naples “Federico II”, Via Cintia 4, I-80126 Naples, Italy

**Keywords:** procyanidins, prodelphinidins, (epi)catechin, antioxidant, food stabilization, polymer reinforcers, structure−property relationships, agri-food byproducts

## Abstract

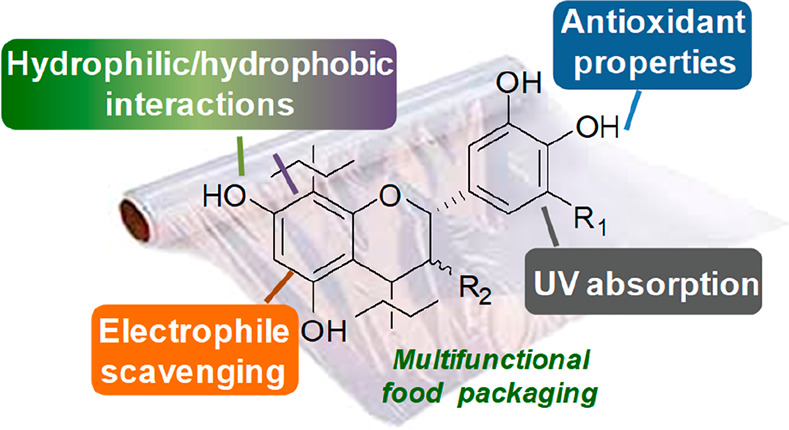

Condensed tannins (CT) have been the focus of increasing interest in the last years as a result of their potent biological properties, which have prompted their use in the food and feed sector as functional ingredients. The possible exploitation of these compounds as multifunctional additives for the implementation of active food packaging has also been recently appreciated. In this perspective, an overview of the structural features, accessible sources, methods of analysis, and functional properties of CT is provided, with the aim of critically emphasizing the opportunities offered by this widespread class of natural phenolic compounds for the rational design of multifunctional and sustainable food packaging materials.

## Introduction

In the past decade, the interest toward natural phenolic compounds has tremendously increased within the scientific community, expanding beyond their established health-promoting effects and encompassing other functional properties that have prompted their application as additives for the implementation of biomaterials, cosmetics, or products designed for the food industry.^1−3^ In this latter context, growing importance has been gained by food packaging incorporating phenolic compounds as active components able to prolong the shelf life of food and/or as stabilizers of the packaging material itself against, e.g., thermal and photo-induced degradation.^4,5^ This applies in particular to phenolic polymers, above all tannins and lignin, which are highly attractive compared to low-molecular-weight compounds in terms of stability, ease of processing, and toxicity.^6^

Starting from this premise, this perspective is focused on the still not fully exploited opportunities offered by a particular class of phenolic polymers, that is condensed tannins (CT), for the implementation of multifunctional and sustainable food packaging. After a short presentation (several comprehensive reviews on these topics are available as quoted below) of the main structural features and sources, with special emphasis on byproducts deriving from the agri-food sector, the functional properties of natural CT will be critically surveyed, followed by a discussion about the emerging technologies and strategies for the design of multifunctional packaging materials.

## Main Structural Features and Sources of Natural CT

**Figure 1:**
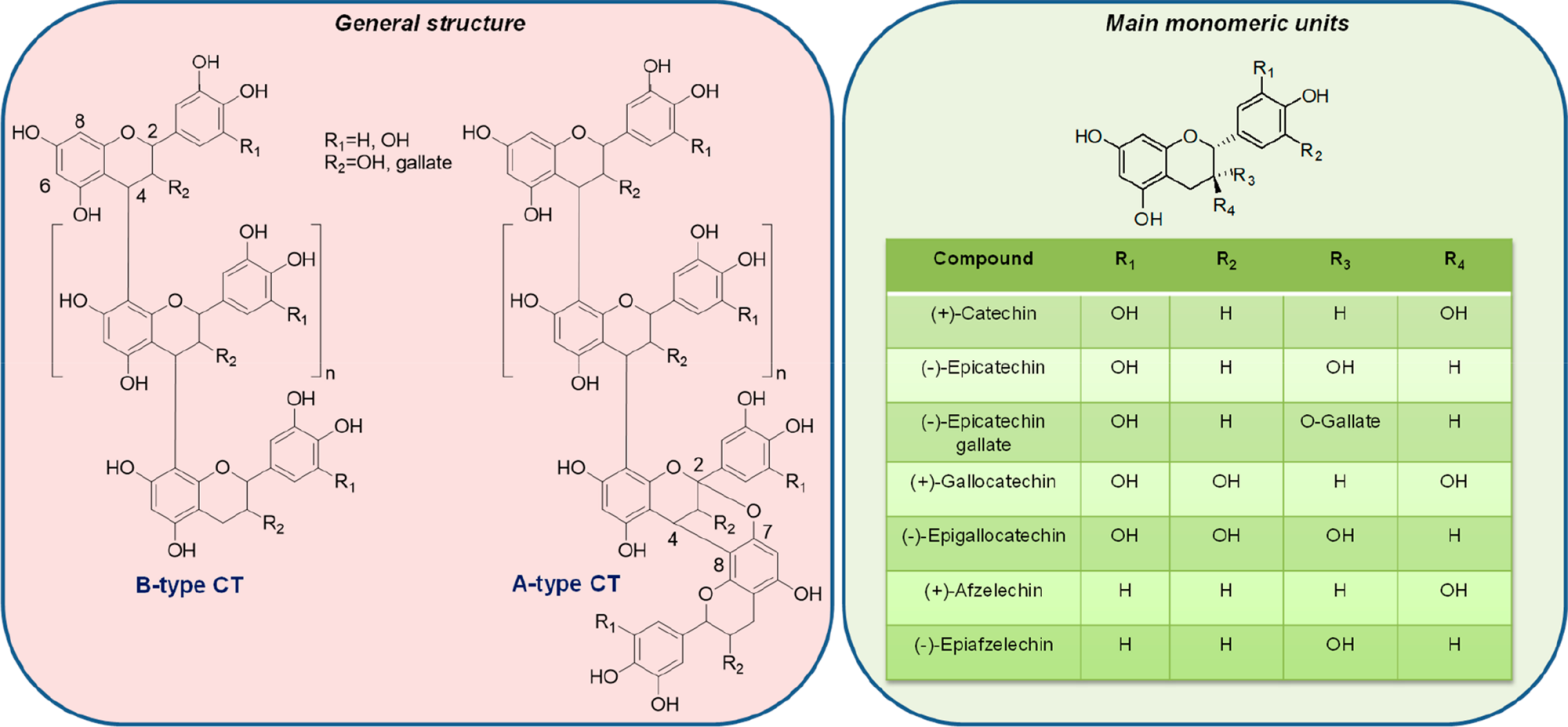
General structure and main monomeric components of natural CT.

Condensed or non-hydrolyzable tannins are chemically heterogeneous oligomers and polymers (molecular weight in the range from 500 to over 20 000 Da) of polyhydroxyflavan-3-ol monomer units linked mainly by C_4_–C_6_ or C_4_–C_8_ bonds (B-type CT). Less widespread are the A-type CT, characterized by the presence of flavanol units doubly linked by C_4_–C_8_ and C_2_–O_7_ or C_4_–C_6_ and C_2_–O_7_ bonds (left panel of Figure 1). CT are often referred to as proanthocyanidins because they can release anthocyanidins upon depolymerization, which occurs only under strongly acidic conditions. Catechin and epicatechin are the most representative monomeric units in natural CT, together with epicatechin gallate and to a lesser extent gallocatechin, epigallocatechin, afzelechin, and epiafzelechin (right panel of Figure 1).^7−10^

CT are considered the second most abundant group of natural phenolic polymers after lignin and are widely distributed in the plant kingdom. The highest concentrations can be found in the bark and heartwood of a variety of tree species, first of all mimosa, quebracho, and oak, but they are also abundant in nuts, fruits, and seeds as well as in leaves, twigs, and stems of some leguminous plants.^6,8,11,12^ Berries in particular are a rich source of CT, together with persimmon, banana, and apples.^7^ High contents of CT have also been determined in cocoa and grape seeds.^7,13^

Given the widespread occurrence and the growing global interest toward green production processes, in the last years the possibility to recover these compounds from low-cost, largely available, and sustainable sources, such as agri-food byproducts, has been intensively investigated.^14−16^ One of the richest sources (up to 25% w/w, of dry matter) of CT is undoubtedly grape pomace, especially grape seeds,^7,13,14,17,18^ followed by walnut and peanut skin,^7,12^ canola hull,^7^ pecan nut and cocoa shell,^7,12,19^ bean seed coat,^7^ buckwheat hull,^7^ and apple peel.^7^ Recently, the possibility to exploit exhausted woods from tannin extraction as a source of high-molecular-weight CT has also been reported.^20^

CT from grape seeds (*Vitis vinifera*), mimosa (*Acacia mearnsii*), quebracho (*Schinopsis balansae*), and pine (*Pynus pinaster*) bark are commercially available.

## Extraction and Structural Analysis Methodologies of CT

Several extraction conditions of CT have been reported in the literature, depending upon the starting materials. Hot water extraction is generally the preferred approach given the low cost and simplicity, although the use of water in combination with organic solvents, mainly acetone, has also been described.^13,21^ The positive impact on extraction yields of enzymes able to degrade the cell wall, thus helping the release of tannins, and advanced technologies, like ultrasound, microwave, and pressure applications, possibly coupled with supercritical fluids or ionic liquids has also been recently reported.^8,21^ Of course, the extraction efficacy is largely dependent upon the extraction conditions, that is, time, temperature, and solid/solvent ratio, as well as the particle size.^21^

As far as structural analysis of CT is concerned, this is a very important issue, given their high structural diversity, which of course affects their functional properties.

**Figure 2:**
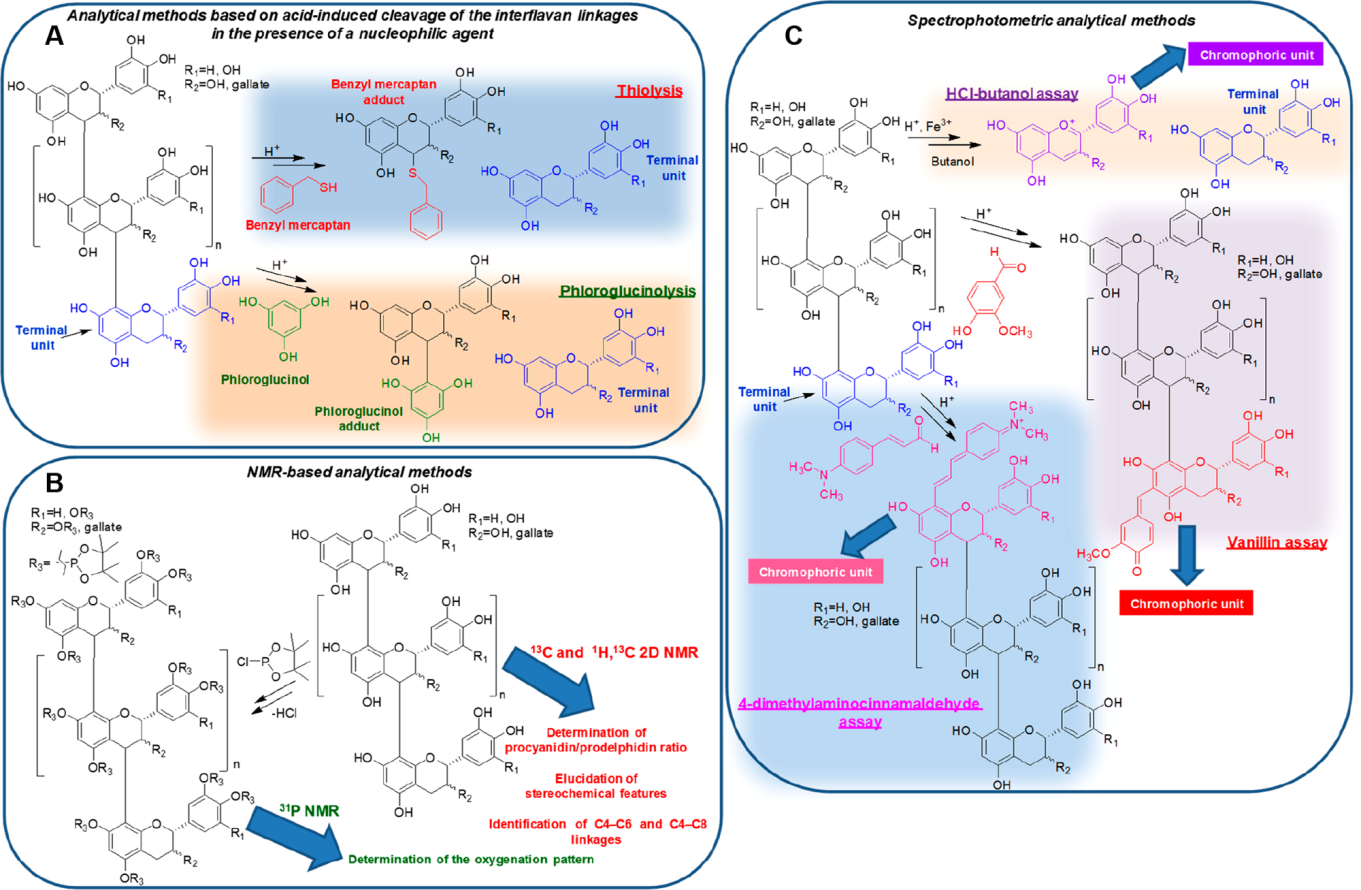
Overview of the main analytical methodologies for structural analysis of CT.

Thiolysis is undoubtedly one of the most commonly employed analytical methodologies.^10,22^ It is based on the acid-induced cleavage of CT into the individual flavan-3-ol subunits, which undergo attack by a thiol (usually benzyl mercaptan), apart from the terminal unit, which is liberated as an underivatized flavan-3-ol (Figure 2A). High-performance liquid chromatography (HPLC) and/or liquid chromatography–mass spectrometry (LC–MS) identification and quantitation of the released units may thus provide structural information on the main monomeric units and the mean degree of polymerization (mDP). Of course, to this aim, reference standards and HPLC response factors are needed. Phloroglucinolysis (Figure 2A), involving the use of phloroglucinol in place of the smelly and lachrymatory benzyl mercaptan, has been proposed as a valuable alternative to thiolysis, although in some cases, it has been found to be less effective in terms of adduct formation yields.^22^ Very recently, an efficient analytical depolymerization method for characterizing CT based on the use of menthofuran as the nucleophilic trapping reagent has been developed.^23^

^13^C nuclear magnetic resonance (NMR) analysis is gradually emerging as a more refined technique for structural analysis of CT, also being the only technique allowing for the distinction between C_4_–C_6_ and C_4_–C_8_ linkages.^10^ In addition, whereas this kind of analysis was initially applicable only to soluble CT, the advent of solid-state techniques, such as ^13^C cross-polarization magic angle spinning (CPMAS) spectroscopy, possibly coupled with a two-dimentional (2D) analysis has significantly contributed to expand its range of applications. Additional structural information may be obtained by derivatization of the CT sample with a phosphorylating agent, such as 2-chloro-4,4,5,5-tetramethyl-1,3,2-dioxaphospholane (Cl-TMDP), followed by ^31^P NMR and ^1^H and ^13^C 2D NMR analyses, as recently reported (Figure 2B).^10,24,25^

Other methodologies allowing for direct analysis of CT include matrix-assisted laser desorption/ionization time-of-flight mass spectrometry (MALDI–TOF MS)^10^ and advanced normal-phase LC, reverse-phase LC, or hydrophilic interaction liquid chromatography (HILIC), possibly coupled with fluorescence detection.^26−29^

Relatively simpler spectrophotometric methods are still widely adopted that allow for the quantification of the amounts of CT in plant materials: these are the HCl–butanol assay and the vanillin or 4-dimethylaminocinnamaldehyde assays (Figure 2C),^10,22,30^ whose response, however, is somewhat unreliable because it is strictly dependent upon the experimental conditions adopted (solvent, acid concentration, time, and temperature) and the CT structure and solubility.

It is clear, however, that multiple characterization methods are needed to adequately analyze and describe the amount and kind of CT present in a sample.

## Chemical and Functional Properties of CT

The peculiar chemical structure makes CT a natural chemical platform not only endowed with intrinsic functional properties but also susceptible of a series of structural modifications allowing for an even wider exploitation of these polyphenols as a sustainable and eco-friendly alternative to fully synthetic compounds. Apart from the century-old application in the leather and fiber dyeing industry, CT are commonly employed as coagulants for environmental applications, adhesives for wood, tires, or concrete, ore flotation agents, anticorrosive chemicals for metals, and flame retardants.^8,21^ They are also used in health-related and cosmetic applications as well as animal feed additives as a result of their antioxidant, cardioprotective, neuroprotective, immunomodulatory, antidiabetic, anticancer, antimicrobial, anthelmintic, antiviral, anti-inflammatory, and biopolymer stabilization properties.^9,31−33^ With regard to the food sector, the possibility to exploit CT as functional additives in polymeric materials to be used in food packaging has been increasingly appreciated (as described in more detail in the following section) in view of the manifold opportunities offered by these phenols.

**Figure 3:**
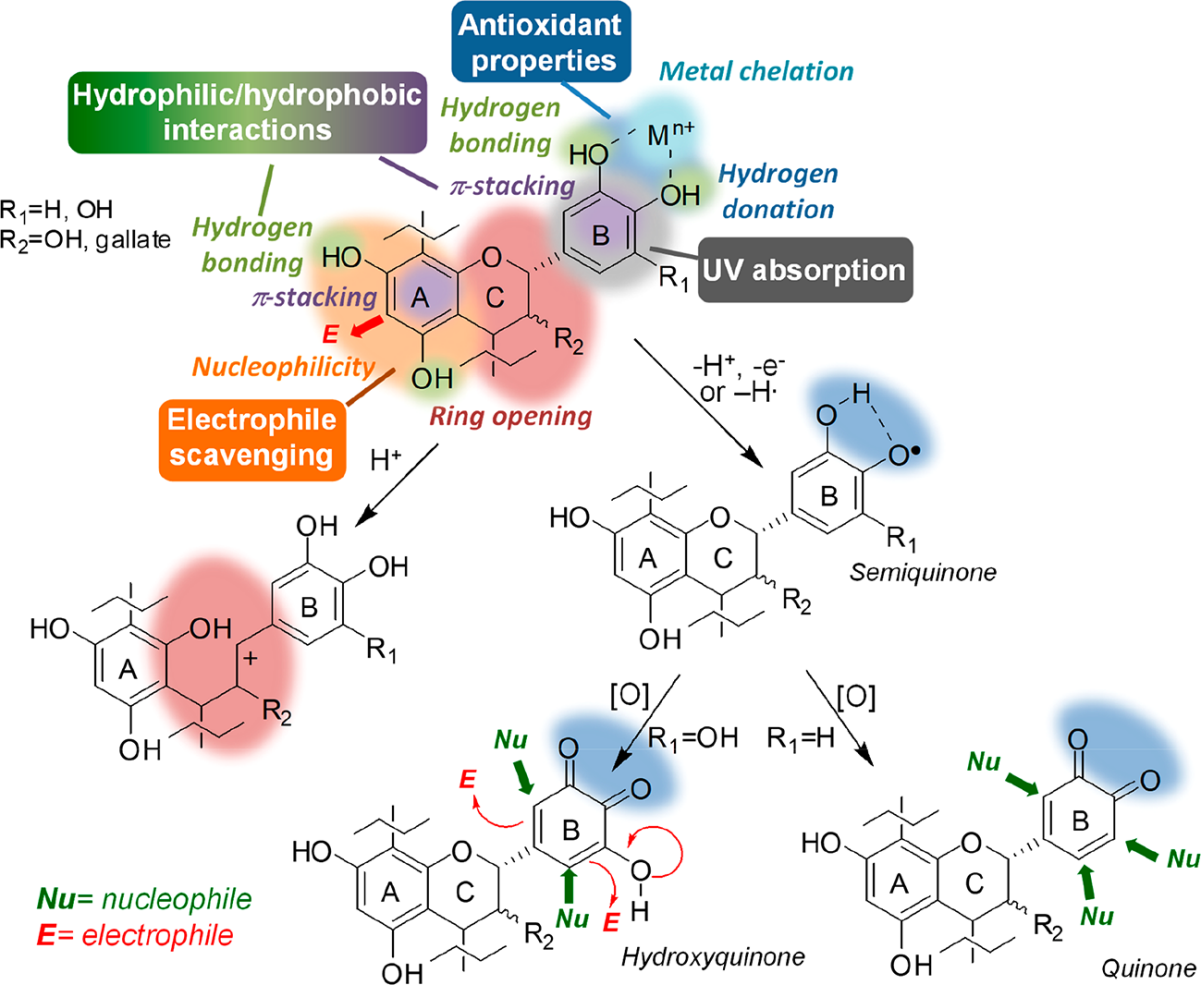
Main chemical properties and reactivity of CT. Bold colored arrows indicate the main sites of reactivity with nucleophilic and electrophilic species.

Multifunctionality is undoubtedly a distinctive trait of CT,^34,35^ and it derives from the peculiar chemical structure of the monomeric components, that is, limiting to procyanidins and prodelphinidins for the sake of simplicity, (epi)catechin and (epi)gallocatechin, respectively. These compounds are composed of a catechol/pyrogallol B ring, a resorcinol-like A ring, and a heterocyclic C ring. The first is mainly responsible for the high antioxidant properties of CT, with the bond dissociation energy (BDE) of the 4′-OH bond being relatively low as a result of the stabilizing effects of the adjacent OH group(s) on the resulting semiquinone radical (Figure 3). Further oxidation of this latter gives rise to electrophilic *ortho*-quinones in the case of (epi)catechin moieties or hydroxy-*ortho*-quinones in the case of (epi)gallocatechin units, which can react as both electrophiles and nucleophiles with a variety of compounds (Figure 3). The catechol/pyrogallol B ring may exert an antioxidant activity also by chelating metal (e.g., iron and copper) ions (Figure 3), which are involved in the initiation of oxidative processes. As far as the A ring is concerned, the high electron density as a result of the *meta* pattern of OH group substituents makes it a very strong nucleophile able to react with several electrophilic species (Figure 3). Under harsher experimental conditions, the heterocylic C ring may also exhibit chemical reactivity, undergoing hydrolysis and ring opening by exposure to strong acids (Figure 3), and autocondensation at acidic or alkaline pH values. Finally, all of the nucleophilic phenolic and aliphatic OH groups of CT can undergo chemical modifications by reaction with proper electrophiles (see below).

Other important chemical features of CT, which are at the basis of their functional properties, are the ability to establish strong hydrogen bonds through the numerous OH groups and the hydrophobicity of the aromatic rings, allowing for π-stacking interactions (Figure 3). The strong ultraviolet (UV) light absorption properties (λ_max_ ca. 280 nm) is another important feature of CT of practical interest (Figure 3).

Knowledge of the chemical properties of CT is fundamental for a full understanding and a rational exploitation of the countless opportunities offered by these versatile compounds in application fields of interest for scientists working in the agricultural and food sector, including notably food packaging.

## Applications and Opportunities Offered by CT in the Food Packaging Sector

**Figure 4:**
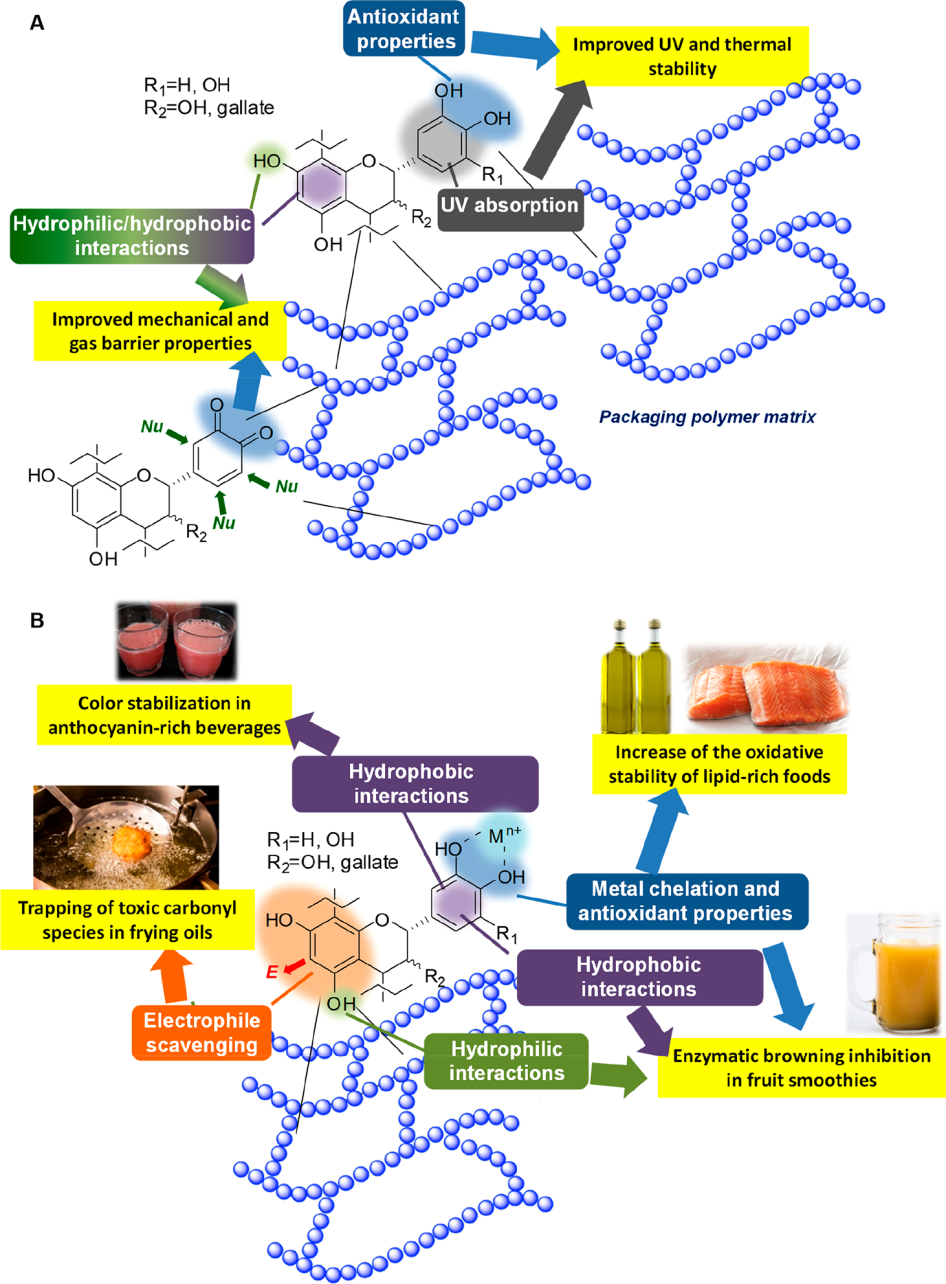
Exploitation of CT chemical properties in food packaging: (A) role of CT for reinforcement of the packaging polymer matrix and (B) role of CT as functional additives able to delay the onset of deterioration processes and prolong the shelf life of food.

As mentioned in the previous section, CT are being increasingly exploited as multifunctional additives in the food packaging sector. Indeed, besides a good biocompatibility and antimicrobial activity allowing for safe counteraction of food bacterial spoilage,^6,9,36^ the peculiar chemical properties of CT open a series of opportunities and provide several advantages for the design and implementation of functional packaging. In particular, two main levels of applications may be envisaged, that is, (1) the use of CT for reinforcement of the packaging polymer matrix in terms of mechanical properties, UV and thermal stability, and gas permeability (Figure 4A) and (2) the use of CT as functional additives able to delay the onset of deterioration processes and, hence, prolong the shelf life of food (Figure 4B).

Crucial for the use of CT as reinforcing fillers for polymers are the efficient antioxidant properties, allowing for the stabilization of materials commonly employed in the food packaging, such as polyolefins (polyethylene and polypropylene) and polylactic acid (PLA), against thermal and/or photo-induced oxidative degradation. The UV-shielding properties of CT of course additionally contribute to the photostability of the polymer. Moreover, the possibility to strongly interact with the polymeric matrix through both hydrogen bonds and, depending upon the kind of matrix, covalent bonds further to oxidation of the B ring and conjugation with nucleophilic groups present in the polymer generally leads to an improvement of the mechanical properties, such as tensile strength, and the water vapor and oxygen barrier properties, which are of paramount importance to keep the food fresh and protect it from, e.g., oxidative deterioration (Figure 4A).

As far as the “direct” effect on the extension of the shelf life of food is concerned, several applications of CT have been proposed, prompted again by their peculiar chemical properties, combined, as mentioned above, with strong antimicrobial and antifungal activities against several foodborne pathogens. The antioxidant and metal-chelating properties of CT have been explored in the food industry to increase the oxidative stability of lipid-rich foods during storage,^9^ whereas the ability to establish π-stacking interactions with aromatic compounds is at the base of their possible exploitation as “co-pigments” able to enhance anthocyanin storage and heat stability by preventing, e.g., water addition to the flavylium ion, thus preserving the color intensity in red wine or fruit- and berry-derived foods and beverages.^19,37^ The possible use of CT to delay enzymatic browning processes in fruit smoothies has also recently been reported, with these compounds being able to inhibit the activity of polyphenol oxidases, such as the copper-containing tyrosinase, by binding to the active site of the enzyme through hydrogen-bonding and hydrophobic interactions as well as chelating the copper ions present at the active site through the *ortho*-diphenolic functionality on the B ring.^2,19,38^ However, it should be taken into account that CT can also act as substrates of polyphenol oxidase and, hence, produce brown pigments themselves;^39,40^ therefore, control experiments should be performed before resorting to this approach. Finally, the addition of CT to frying oils to improve their quality through removal of toxic carbonyl species has also been proposed on the basis of the high nucleophilicity of the A ring, leading to the formation of carbonyl–phenol adducts^41^ (Figure 4B).

Several reports on the use of CT as active components in food packaging have recently appeared in the literature. Incorporation of CT is generally achieved through extrusion,^19,42^ solvent casting,^19,43^ or vacuum filtration, followed by dehydration,^44,45^ without the need for chemicals to covalently link the antioxidant to the polymer, although in some cases, the use of plasticizers, such as glycerol, has been reported.^46−48^ Scanning electron microscopy (SEM) and transmission electron microscopy (TEM) are routinely employed for the morphological characterization of the functionalized polymeric material,^19,44,45,48,49^ whereas the optical transmittance profiles and color of the films can be recorded and defined by use of an ultraviolet–visible (UV–vis) spectrophotometer and colorimeter, respectively.^42,44,45,48^ On the basis of the UV absorption properties of CT, UV–vis spectroscopy, possibly coupled with gravimetric tests,^49,50^ can also be used to monitor the release of the additive from the film when in contact with the food matrix or reference solvents.^19,47^ As far as the effects of CT on the mechanical properties of the polymeric films are concerned, tensile tests are generally performed,^19,44−50^ along with dynamic mechanical analysis.^46^ Other characterizations of the functionalized films generally involve gas (e.g., oxygen, water vapor, air, and carbon dioxide) permeability,^19,44,47−49^ water contact angle,^44^ and water uptake/swelling measurement.^46,50^ The effective incorporation of CT and the kind of interactions between the polymer and the additive can be easily determined by attenuated total reflectance Fourier transform infrared (ATR–FTIR) spectroscopy and/or thermogravimetric analysis (TGA).^19,43−45,48,49^ TGA is also used to characterize the thermal stability of the films,^42,48^ whereas the photo-oxidative stability can be determined by ATR–FTIR spectroscopy and tensile tests further to light exposure.^42^ The antioxidant properties acquired by the functionalized films can be straightforwardly evaluated by means of the widely used antioxidant assays 2,2-diphenyl-1-picrylhydrazyl (DPPH), ferric reducing antioxidant power (FRAP), and oxygen radical absorbance capacity (ORAC) assays,^19,43−45,47,49^ whereas for the determination of the antimicrobial activity, the agar plate diffusion method^43,47,48^ or optical density measurement^50^ are generally applied. Biofilm formation inhibition activity can also be determined using the microplate assay^50^ or confocal laser scanning microscopy.^47^

As a remarkable example of the multifunctionality that may be imparted by CT to food packaging materials, PLA films incorporating CT from pecan nut shell exhibited improved mechanical properties and thermal and photo-oxidative stability, along with antioxidant and enzymatic browning inhibition activities.^19,42^ The same tannins have also been exploited for the implementation of antimicrobial and antioxidant whey protein-based edible films with excellent gas barrier properties.^47^ Cross-linking interactions between CT from pine bark and proteins have been demonstrated in soy protein isolate films, resulting in improved thermal stability, tensile strength, and antioxidant activity and decreased water vapor and oxygen permeability.^51^ The combination of cellulose nanofibrils and CT from *A. mearnsii* or a commercial quebracho tannin extract has been reported for the development of functional films with prolonged antioxidant effects and selective absorption of UV light while maintaining optical transparency in the visible range or with enhanced air-barrier properties.^44,45,49^ Along the same line, the incorporation of commercial CT in chitosan films remarkably increased the tensile strength and thermal stability, improved the antioxidant and antimicrobial activities, and significantly reduced the oxygen permeability and UV–vis light transmittance.^48^ Chitosan-based CT composite films for cheese or salmon packaging with high antioxidant activity, bacteriostatic properties, and, in some cases, also pH responsiveness have been very recently reported too.^43,50^

Some CT-functionalized materials for food packaging applications have also been patented.^52−54^

## Improving CT Properties by Chemical Derivatization

Modification of the native structure of CT by chemical derivatization has been increasingly appreciated as a strategy to overcome some drawbacks, like low solubility and too high or too low reactivity that hamper their full exploitation, as well as to introduce or selectively modify the chemico-physical properties of CT with the aim of expanding the range of potential utilization. As an example, acylation or alkylation of the phenolic groups has been exploited as a strategy to increase CT thermal stability and solubility in nonpolar solvents and, hence, improve their performance in materials science.^55^ On the contrary, sulfonation, sulfation, or sulfitation could be applied to improve the solubility of, e.g., quebracho tannins in polar solvents.^55^ The extent and site of OH group derivatization is strictly dependent upon the reaction conditions adopted and the structure of the starting CT. In general, such modifications result in a lowering of CT reactivity, but an improvement of some biological properties, including the bacteriostatic power and the biodegradability, may be attained.^55^

## Challenges and Perspectives

The profile of CT drawn in this perspective has shown how these compounds may offer unrivalled opportunities as natural, eco-friendly, and sustainable additives for the design of both an active and resistant material for food packaging. There are, however, some critical issues in the approaches thus far pursued that should be taken into account in future studies.

- Chemical modifications of CT for application in the food sector should be achieved under food-grade conditions and should not lead to alterations in their biocompatibility. To assess the feasibility and effects of such derivatizations, monomeric units of CT, such as catechin and epicatechin, could usefully be employed. The chemical modifications of these less structurally complex and commercially available compounds could be straightforwardly characterized by conventional spectroscopic and spectrometric methodologies.
- When CT recovered from agri-food byproducts are aimed to be used, possible variability in the composition of different lots from the same source should be considered; this may derive from several factors, including the geographical areas of harvesting of the plant source, the manufacturing process, and the storage conditions. At least the amounts of CT in each new extract should be determined by a rapid spectrophotometric assay before further use.
- Physical inhomogeneity of the sample in terms of the particle size distribution is expected to heavily affect the performance of CT when dispersed into the polymeric matrix for applications in food packaging. Ball-milling treatments followed by sieving may allow for a material to be obtained with a well-defined granulometry.

Beside these limitations that could be overcome by technical solutions, use of CT in food packaging is expected to expand in the future based on a variety of considerations: first of all, the socioeconomic impact. Indeed, CT, particularly those deriving from agri-food byproducts, are expected to have a positive impact on both the environment and daily lives of people. Moreover, the possibility to reduce food loss through the recovery of food wastes and byproducts meets the circular economy and sustainability goals.

As a final remark, a full exploitation of CT based on the development of rational strategies for the implementation of multifunctional packaging should be rooted in a deep knowledge of the structure–property relationships underlying the complexity of natural phenolic compounds.
